# Comparing Synchronous and Asynchronous Remotely Delivered Lifestyle Interventions: Protocol for a Randomized Noninferiority Trial

**DOI:** 10.2196/65323

**Published:** 2024-12-19

**Authors:** Sherry Pagoto, Ran Xu, Richard Bannor, Christie Idiong, Jared Goetz, Denise Fernandes

**Affiliations:** 1 Department of Allied Health Sciences University of Connecticut Storrs, CT United States

**Keywords:** obesity, lifestyle intervention, digital health, weight loss maintenance, engagement, mobile phone

## Abstract

**Background:**

Remotely delivered lifestyle interventions have emerged to increase the reach and accessibility of traditional interventions that involve numerous in-person visits. Remote interventions can be delivered synchronously via videoconference software or phone or asynchronously via online platforms. Asynchronously delivered interventions are convenient and flexible in that they allow people to participate at any time and as such, they may be more sustainable. Evidence for asynchronous interventions is needed given their potential for convenience and sustainability, which may have implications for weight loss maintenance.

**Objective:**

This is a randomized noninferiority trial comparing 2 remotely delivered lifestyle interventions: one that is delivered synchronously via videoconference meetings and one that is delivered asynchronously through private Facebook (Meta) groups. We hypothesize that the percent weight loss difference between conditions at 6 and 12 months will be less than 2% and that the asynchronous condition will cost less to deliver per pound lost. We also hypothesize that engagement will be higher in the asynchronous condition at 12, 18, and 24 months and that the asynchronous condition will have greater weight loss at 24 months.

**Methods:**

We will randomize 328 participants with overweight or obesity to a remotely delivered lifestyle intervention that is delivered either synchronously or asynchronously. Delivery of the synchronous lifestyle intervention will be via videoconference group sessions, whereas the delivery of the asynchronous lifestyle intervention will be via private Facebook groups. The lifestyle intervention in both conditions is based on the Diabetes Prevention Program. The intervention goals are to lose 5%-10% of baseline weight and to work toward 300 minutes per week of moderate intensity physical activity. The core intervention will last for 12 months and be led by counselors in each group. This will be followed by a 12-month maintenance phase to be led by participant volunteers from each group. Participant engagement and weight loss maintenance will be assessed during this phase. The primary outcome is mean percent weight loss at 6 and 12 months. The noninferiority margin for differences in weight loss between conditions is 2% at both 6 and 12 months. We will model percent weight loss at 6 and 12 months using general linear regression models with the intent-to-treat sample. Secondary outcomes include engagement, collective efficacy, cost, and weight loss at 18 and 24 months.

**Results:**

The funding period began on August 17, 2023, and the study was approved by the University of Connecticut Institutional Review Board on August 17, 2023. Participant recruitment will begin December 2024 and the intervention will begin February 2024.

**Conclusions:**

If hypotheses are confirmed, this work will provide evidence that asynchronously delivered remote interventions are as efficacious as synchronously delivered ones and more sustainable such that people will engage in them longer and retain more weight loss for less cost.

**Trial Registration:**

ClinicalTrials.gov NCT06393725; https://tinyurl.com/4kzzwkc9

**International Registered Report Identifier (IRRID):**

PRR1-10.2196/65323

## Introduction

Obesity, a significant risk factor for type 2 diabetes, affects 39% of US adults [1,2]. Lifestyle interventions such as the Diabetes Prevention Program (DPP) have shown strong efficacy for producing weight loss, but they have many barriers to participation and are not sustainable long term even though obesity is a chronic condition [3]. Technology allows us to provide lifestyle interventions to patients with fewer barriers and possibly greater sustainability. Technology-delivered interventions utilize online platforms, videoconferencing, or mobile apps to deliver counseling, peer support, and multimedia content to patients. They allow people to participate from anywhere they can access the internet. Technology-delivered lifestyle interventions have been done synchronously via online meetings or asynchronously via messaging on apps or other platforms [4]. The literature lends few insights as to the comparative efficacy of different technology-based modalities for the delivery of lifestyle interventions.

A systematic review of 21 technology-delivered lifestyle interventions based on the DPP revealed a mean percent weight loss at 15 months of about –3.98% and those with moderate to high methodological quality produced about –3.40% [4]. Notably, technology-delivered interventions that included human counseling produced greater weight loss than fully automated programs. Only 3 trials compared digital to in-person lifestyle interventions. In one of those trials, investigators had to pivot from in-person visits to videoconferencing visits during the pandemic and found that weight loss did not differ by modality [5]. However, participants were not randomized to the conditions. In another trial, investigators randomized 43 participants to in-person or videoconference lifestyle interventions and found no weight loss differences between groups, but they were not powered to establish noninferiority [6]. A third trial that was powered for noninferiority compared in-person to an asynchronous technology-delivered intervention and found that the in-person intervention resulted in significantly more weight loss at 6 months, but not at 12 months [7]. A gap in the literature is how asynchronous delivery compares with synchronous delivery when both interventions are delivered via technology. Asynchronous lifestyle interventions, by not requiring that participants be available to attend regularly scheduled meetings, offer a convenient alternative to synchronous interventions delivered via videoconference.

Asynchronous interventions allow counselors and participants to engage individually through messaging or in groups via discussion threads. One randomized trial tested the efficacy of an asynchronous lifestyle intervention delivered via a commercial mobile app (Noom) that involved asynchronous messaging with coaches and a group of peers. Intervention participants lost a significantly higher percentage of weight than those in a no-treatment control group at 6 months (mean=–3.69% vs –0.15%) and 12 months (mean=–2.54% vs 0.33%) [8]. Several pilot trials have established the feasibility and acceptability of asynchronous technology-delivered lifestyle interventions [9-14], including one trial of rural adults that compared the feasibility of an asynchronous lifestyle intervention delivered via Facebook to a synchronous version delivered via videoconferencing [10]. It found high rates of retention, satisfaction, and dietary self-monitoring compliance in both groups with no differences between groups. Weight loss at 6 months was not different between groups but this trial was not powered for noninferiority. No fully powered weight loss trials have compared an asynchronous technology-delivered lifestyle intervention to a synchronous one. However, a randomized trial of adults with depression compared an asynchronous technology-delivered depression therapy to a synchronous video teletherapy condition [15]. In the asynchronous condition, participants could send messages to their therapist at any time and receive daily replies. In the synchronous condition, participants met with their therapist once weekly for a 45-minute video teletherapy session. Noninferiority was established for the asynchronous condition in depression, anxiety, and functioning outcomes and participants found this modality highly acceptable. More research is needed to establish the efficacy of asynchronous technology-delivered lifestyle interventions given that many people with obesity may not be able to participate in programs that require attendance at scheduled meetings either in person or remotely.

Since the dawn of email, SMS text messaging, and social media, asynchronous communication has become ubiquitous in everyday life. A survey of 1300 US adults found that 77% said the feature they use most on their smartphone is texting, 64% said social media, 48% said email, and only 32% said phone calls [16]. Social media use, which is comprised of asynchronous exchanges, is prevalent in the United States. The Pew Internet Research Survey reported that 72% of US adults have at least one social media account [17]. On average, social media users spend 147 minutes a day (~2.5 hours) on social media sites [18]. Because so much human communication is asynchronous, research is needed to explore how to deliver health care services through asynchronous communication technologies. Rather than inventing a new asynchronous technology to deliver a lifestyle intervention, we have been using commercial social media platforms as a means to “meet people where they are” given how much time people spend on these sites [13]. This sidesteps the expense of developing a novel platform and trying to get people to log into and use it, especially when their attention is absorbed by popular social media platforms.

An advantage of asynchronous interventions delivered via social media platforms is the potential for sustainability, which, in this context, we define as the length of time the intervention group remains intact. To the extent that the group remains intact, members can continue to receive support and access resources. A major problem with synchronous lifestyle interventions, whether delivered in-person or via technology, is that the intervention group disbands when the intervention is over, thus participants lose the support and resources the group provided. Unsurprisingly, weight regain is routinely observed in the period following lifestyle interventions [19]. Asynchronous lifestyle interventions delivered on commercial social media platforms like Facebook may be more sustainable because staying in the group requires no effort on the part of participants and the intervention content is still available to them as long as they remain in the group. Participants already use Facebook multiple times a day and will continue to after the program, which means the space created on Facebook can continue to be used by participants long after the program ends. In one pilot feasibility trial of a lifestyle intervention conducted in private Facebook groups, participants were randomized to a group of 40 participants or a group of 94 participants and after the 3-month intervention, the counselors exited both Facebook groups, but the groups remained open for 1 year and were each led by a participant volunteer [13]. Engagement in the large group continued through month 8 and then dropped off, whereas in the small group, engagement ceased after month 3. Furthermore, the larger group (n=94) produced nearly 5-fold greater engagement than the small group, which was much larger than the proportional difference in group size. The findings reveal that asynchronous groups will continue to engage after an intervention ends, even in the absence of an interventionist, and larger groups appear to engage more and for a longer time than smaller groups. Research is needed to examine if greater group engagement following an asynchronous lifestyle intervention is accompanied by greater weight loss maintenance.

The current study is a noninferiority randomized controlled trial testing whether weight loss following a yearlong asynchronous lifestyle intervention conducted in a private Facebook group is noninferior to that of a synchronous lifestyle intervention conducted via videoconference meetings. Due to the ease of engaging in asynchronous groups, we hypothesize that the asynchronous group will have greater engagement than the synchronous group, which requires participants to attend meetings to engage with the intervention. We also hypothesize that the asynchronous condition will cost less per pound lost to deliver. After the 1-year intervention, the counselors in each group will turn group leadership over to a participant volunteer for the subsequent year. We hypothesize that the asynchronous group will engage more and for a longer period of time than the synchronous group and thus retain more weight loss at 18 and 24 months. The conceptual foundation for this hypothesis lies in Bandura’s concept of collective efficacy, which is when members of a group feel connected to their group and believe that the group shares their goals and abilities [20]. In groups with high collective efficacy, group members are empowered to work collectively to solve problems and this facilitates greater success toward goals among the group as a whole. We hypothesize that members in the asynchronous condition, by virtue of having more opportunities to engage (ie, 24/7) will experience higher collective efficacy than the synchronous condition where interactions are restricted to 22 group meetings. Furthermore, we hypothesize that the asynchronous condition will have greater engagement in the postintervention period because it is easier to engage there than it will be in the synchronous condition where engagement will depend on (1) how reliably the volunteer group leader arranges regular videoconference meetings and (2) the availability of group members to attend those meetings. In the asynchronous condition, engaging in the postintervention period entails very little effort and is not as heavily reliant on the volunteer group leader, since any member can post at any time. We suspect collective efficacy may decline in the synchronous condition during the postintervention period due to these participation barriers. We will measure collective efficacy to test our hypothesis that the asynchronous condition will have greater collective efficacy by the end of the 1-year program and the 1-year peer-led maintenance phase than the synchronous condition.

## Methods

### Study Design

The proposed design is a randomized controlled noninferiority trial. We will randomize 328 adults who are overweight or obese to either an asynchronous or synchronous condition. Participants in the asynchronous condition will receive lifestyle counseling via a private group on Facebook. Participants in the synchronous condition will receive a lifestyle intervention via videoconference group meetings. Two waves of 164 participants will each be randomized into two groups of 82 participants. Content in both conditions is based on the DPP Lifestyle Intervention [21]. Measurements will be taken at baseline, 6, 12, 18, and 24 months. Percent weight change at 6 and 12 months is the primary end point. Secondary end points include engagement, collective efficacy, cost, and sustainability, defined as engagement in the year-long postintervention period.

### Study Population

To be eligible, participants must be between the ages of 18 and 65 years, have a BMI between 27 and 45 kg/m^2^, have Bluetooth or Wi-Fi connectivity at home (for scale), go on Facebook at least 5 days per week over the past 2 weeks, and own a smartphone. Participants must not be pregnant, lactating, or have plans to during the study period; have bipolar disorder, substance abuse, psychosis, bulimia, binge eating disorder, or severe depression; have had bariatric surgery or plans to during the study; taking medication that affects weight, lost ≥5% of weight in past 6 months, participated in another weight loss program or plans to during the study; have chronic pain or a medical condition that interferes with the ability to exercise; have type 1 diabetes; be unable to walk one-fourth of a mile unaided without stopping; or use nicotine via cigarettes or vaping on a weekly basis.

### Recruitment

Participants will be recruited via Research Match [22] and ads posted in university listserves, social media, and Craigslist throughout the United States. To attract men who are typically underrepresented in weight loss trials [23], we will use targeted ads as well as recruit on Reddit, where 74% of users are male. To attract racial and ethnic minorities who are typically underrepresented in weight loss trials, we will use targeted ads. We aim to recruit a sample that is 30% (98/328) minorities and 50% (164/328) male. Participants clicking on the recruitment ad will complete electronic consent and a screening survey.

### Onboarding Webinar

Interested candidates who are eligible will complete an online baseline survey and attend a 1-hour study webinar where study staff members will use a Methods-Motivational Interviewing approach to help participants understand the scientific rationale of the trial design, research questions, and methods [24]. This provides participants with a comprehensive understanding of the commitment entailed in trial enrollment. This helps set clear expectations for participants (eg, transparency about the length of assessments), explain the scientific rationale for procedures (eg, randomization and impact of dropouts on conclusions), diffuse ambivalence about research participation using motivational interviewing, increase research literacy, build trust, and make explicit commitments to self and trial. We will also ask participants at this time to not engage with one another outside of the study, including by phone, email, social media, in person, and so on. We will explain the scientific rationale for this request as well as the importance of respecting each other’s privacy by not reaching out to other study participants in ways that participants have not consented to. Upon completion, participants will be mailed a Wi-Fi scale and given log-in info for the scale so we can record weight for assessments. Participants will receive US $40 for completing the baseline assessment.

### Randomization

After participants complete the orientation webinar and set up their scale, they will be randomized 1:1 to the two conditions in randomly permuted blocks of size 4 and 6 using the ralloc program in Stata (StataCorp) [25]. We will stratify randomization by sex and baseline BMI (27-34 and 35-45 kg/m^2^).

### Intervention Conditions

#### Asynchronous Lifestyle Intervention

Two interventionists will each run a group in each condition to balance the conditions by the interventionist. The DPP lifestyle intervention will be delivered in a counselor-led Facebook group with twice daily preprogrammed posts as in our previous studies [12,23,26,27]. All Facebook groups will be on the “private” setting, which means only group members can see the group and its content. Each week’s content is based on the corresponding module of the DPP. The DPP assigns participants the goals of (1) calorie tracking to achieve a calorie goal based on the amount needed to lose 1-2 pounds per week, (2) developing a heart-healthy diet, (3) engaging in 300 minutes per week of moderate intensity exercise, and (4) engaging in strength training twice or more a week. On Mondays, the counselor posts 2 goals for the group to work on that week including one diet goal (eg, reduce added sugar) and one exercise goal (eg, add 15 minutes of moderate intensity exercise on 3 days) to help participants progress toward the overall program targets. On Fridays, the counselor posts a weigh-in post asking participants to reply with their weight change in pounds for the week. This ensures participants are weighing themselves weekly and allows an opportunity for problem solving for those not losing weight. Goal accountability happens each Sunday when the counselor posts asking participants to report how they did on the weekly goals. In between these recurring posts are posts that reflect the DPP module for the week. Many posts contain links to a Pinterest page where we house myriad recipes, meal plans, and workouts tailored to a wide range of dietary preferences (eg, vegetarian) and cultural influences (eg, African American and Latinx). We also include images in our posts that represent individuals from diverse backgrounds by gender, race and ethnicity, marital status, and sexual orientation. Finally, participants can send their counselor private messages.

#### Asynchronous Peer-Led Maintenance Phase

At the end of the 12-month intervention, counselors will query their groups for 2 volunteer moderators to take over the leadership role of each group for the next 12 months. In a previous pilot trial, we were successful in getting volunteer moderators at first request in both groups [13]. In that trial, we did not give volunteers specific guidance on what to post or how to run the group, and we discovered that under those conditions, volunteer moderators continued the weigh-ins but otherwise relied on group members to post in the group. This time we plan to give the volunteer moderators a library of 182 posts (based on the DPP protocol) that they can draw from to start conversations. The library will include goal-setting posts, weigh-in posts, problem-solving posts, goal accountability posts, and additional content that emphasizes lessons learned from the DPP. We will also arrange a 30-minute orientation call with each volunteer moderator to discuss the posts, give them guidance on how to moderate a Facebook group, send them a 19-minute video produced by Facebook on how to run a Facebook group (including guidance on privacy), advise them to post daily and encourage group members to post updates about their progress and ask the group their questions, and finally, we will advise them on how to secure a replacement moderator if and when they no longer want to moderate the group. Moderators may use the library as they wish and post whatever they or the group prefers. The counselors will exit the group when the maintenance phase commences. Study staff members will remain in the group but will not post or engage unless any activity occurs that could indicate a breach of confidentiality or any other type of harm. Neither occurred in our previous pilot. Participants will be informed that study staff members will extract engagement data from the group during this phase.

#### Synchronous Remote Lifestyle Intervention

The DPP lifestyle intervention will be delivered by a counselor in weekly videoconference sessions. The 16-session Core of the DPP Lifestyle Intervention will be delivered over 6 months (90 mins/meeting) followed by monthly meetings for 6 months (22 meetings total) [21].

#### Synchronous Peer-Led Maintenance Phase

At the end of the intervention period, the counselors will query their groups for 2 volunteer moderators to take over the leadership role of each group for the next 12 months. The leadership role entails hosting the videoconference sessions. We will give the group leaders access to a videoconference account so they may host sessions without a cost. As in the asynchronous condition, we will provide the group leaders with additional content from the DPP to use in the groups if they so choose. We will also arrange a 30-minute orientation call with each volunteer group leader to discuss the content, give them guidance on how to lead a group, and advise them on how to secure a replacement group leader if and when they no longer want to lead the group. Group leaders may use the content we provide as they wish or run the group in whatever way they and the group prefer. The counselor will not attend the group meetings; however, a study staff member will attend to record attendance and run the transcription software, but they will not engage unless any activity occurs that could indicate a breach of confidentiality or any other type of harm. Participants will be informed that study staff members will save the chat data and use transcription software to record the group conversation during this phase so that this data can be used for research purposes.

#### Follow-Up Assessments

At 6, 12, 18, and 24 months after baseline, weight data will be collected from Wi-Fi scales and participants will complete online surveys. Compensation is US $40 at 6 months, US $50 at 12 months, US $40 at 18 months, and US $50 at 24 months. Participants who complete all of the follow-ups will receive a bonus of US $50.

### Measures

#### Weight

Weight will be gathered from the Wi-Fi scales we provide to participants as in our previous research [28]. In the event that Wi-Fi is not working, participants will be asked to send a photo of their weight on the scale. Participants will be advised to weigh themselves in the morning, without clothing, before eating and after voiding. We will download weight data from participants’ scales at each time point. We will calculate absolute weight change in pounds and percent weight change.

#### Engagement

Because the nature of engagement in each condition is different, the metric by which we will compare conditions will be word count, defined as total spoken or typed words. In the asynchronous condition, we will manually extract engagement data from Facebook, including posts, replies, reactions (eg, like, love, angry, and haha), poll votes, and private messages to the counselor. Engagement data will be extracted during the intervention phase and the peer-led maintenance phase. The total word count will include the total word count from replies and posts. For Facebook reactions (eg, hitting a like button), the word used will be the type of reaction button used (like, love, laugh, anger, sad, and care). For poll votes, the words included will be those in the option they voted for in the poll. Facebook puts a 24-character limit for each answer option in a poll and every effort will be made to balance the word count across poll options. Each participant’s engagement will be exported into a separate file, and then Pennebaker’s word counting software (Linguistic Inquiry and Word Count, LIWC-22), which has been validated for research purposes [29], will be used to determine the total words typed in the group by each participant and the proportion of words that reflect 4 dimensions of language (analytical thinking, clout, authenticity, and emotional tone). Analytical thinking refers to the degree to which the language used is formal and logical and reflects hierarchical thinking patterns. Language that is low in analytical thinking is more intuitive, personal, friendly, and warm. Clout refers to the language that expresses social status, confidence, or leadership. Authenticity refers to language that is honest, spontaneous, and lacking in social inhibition, and it is characteristic of conversations between close ties or friends. Finally, emotional tone reflects the number of positive emotion words (positive tone) and negative emotion words (negative tone) used.

In the synchronous condition, we will use Webex videoconference software to record and transcribe the group meetings. Then a research staff member will compare the transcript to the video for accuracy and make edits to the transcript accordingly. Because participants might use the chat to ask questions or make comments, we will also save the chats so this data can be included in the word count for each participant. Each participant’s words spoken and typed will be exported into a separate file and analyzed with LIWC-22 as well.

#### Collective Efficacy

Participants will complete the Online Collective Efficacy Scale, a 35-item validated scale designed to assess the degree to which members of an online group feel they are active contributors to a high-functioning group [30]. This measure has three subscales, which are (1) social presence, (2) engagement, and (3) collaboration and augmentation.

#### Cost

We will systematically track costs associated with the delivery of both intervention conditions, capturing information on the costs that would be required to implement each intervention in practice (ie, outside a research context) [31]. Intervention costs will be distinguished from costs associated with research and development (eg, recruitment). We will create an accounting system that captures administrative and intervention time for both conditions. We will use cost-capture templates to evaluate staff members’ time. National salary data will be used to calculate costs including administration (eg, staff members’ time to schedule intervention posts) and intervention delivery (eg, counselor time) costs.

Counselor time will be measured in each condition so that cost can be calculated. In the asynchronous condition, counselors will be given a study phone to use exclusively for counseling and will create a unique Facebook account for the study and only use it for their designated group. The time a user spends on Facebook each day can be found in settings under “Your Time on Facebook” [32]. Counselors will be asked to enter the minutes spent each day into a Research Electronic Database Capture (REDCap, Vanderbilt University) database each week. In the synchronous condition, counselors will enter the minutes spent preparing for and conducting the group meetings in a REDCap database each week.

#### Contamination

At 1 year and 2 years, survey items will assess whether participants engaged with each other outside of their assigned intervention modality, including in person, phone, email, social media, videoconference, or otherwise. They will be asked how many participants they engaged with through these alternative means and how many contacts they had. We will also assess if they engaged in any form of online or in-person weight loss programs so that this can be controlled for in the analyses.

### Sample Size Estimation

We powered this trial to detect noninferiority for the primary outcome, percent weight loss at 6 and 12 months. We determined the sample size using methods developed for noninferiority trials [33]. In this noninferiority trial, the null hypothesis is that the asynchronous condition is inferior to the synchronous condition and the alternate hypothesis is that the asynchronous condition is noninferior to the synchronous condition. “Not inferior to” is defined by the noninferiority margin, δ. Here, we set δ=2%, based on a clinically meaningful difference in mean weight loss between the two conditions. Thus, adequate power for clinical noninferiority requires a sample size such that there is a better than 90% probability that the lower limit of the CI lies above –δ, if the true effect size is zero or above. We will use an SD of 5.5% based on our previous trial [7]. With α=.05, and δ=2%, we have 90% power to conclude that the asynchronous condition is not inferior to the synchronous condition with 131 participants per arm. Assuming 20% attrition, we will enroll 328 participants (164 per arm). Weight loss maintenance will be tested in a longitudinal design, assuming 5 time points (including pretest, 6, 12, 18, and 24 months) clustered within individuals. From the conservative perspective of the inferiority effect size, δ=2%, SD 5.5%, with α=.05, and intraclass correlation coefficient (ICC) 0.6, this trial will have power of 97.6% to yield a statistically significant result with a sample size of 328 participants.

For engagement, we have 80% power to detect differences of ≥0.26 SDs in engagement (word count) between conditions. With 164 participants available per arm (N=328) and α=.05, we have 90% power to detect differences in mean cost per participant of 0.36 SD. For example, if the SD for cost is US $100, then we have 94% power to detect differences in mean cost per participant of US $35. In our previous noninferiority trial comparing a synchronous in-person lifestyle intervention to an asynchronous remote lifestyle intervention, the difference in cost per participant was US $82.66 [7]. We suspect the difference will be less in this trial because travel costs contributed to the difference in our previous trial which used an in-person condition.

In terms of engagement during the Peer Led Maintenance Phase, we have 80% power to detect differences of ≥0.79 SDs in engagement (word count) between conditions.

### Analytic Plan

Reporting and data analyses of this trial will follow the recommendations laid out in the study by Piaggio et al [34]. We will use an intention-to-treat approach, meaning all randomized participants will be included in the model in their originally randomized conditions. We will evaluate the comparability of baseline characteristics by condition. If groups differ on any variables, those variables will be used as covariates in the primary analyses. Other preliminary analyses will include assessing patterns of missing data, dropout rates, distributional properties of dependent measures, and correlations among outcome measures. Attrition in weight loss studies may not be random [35,36]. We will perform a series of sensitivity analyses to understand the extent of potential bias by assuming the subjects who dropped out are missing completely at random (ie, independent of the outcome), are responders to the intervention, or are nonresponders to the intervention. We will have quality checks to make sure our missing data is minimal but if we have more than 5% data missing, we will use multiple imputations [37,38].

We will model percent weight loss at 6 and 12 months using a general or generalized (depending on outcome distribution characteristics) linear regression model framework, with percent weight loss as the dependent variable and intervention condition as the independent variable, and include any unbalanced participants’ characteristics as covariates. This model will provide a statistical test of the intervention effect and the estimated coefficient, and the estimated CI will provide the estimate of the intervention effect. Our analytic approach aims to test whether the asynchronous condition is not appreciably worse than (ie, not inferior to) the synchronous condition by our a priori inferiority margin of 2%. The effect size reveals clinical noninferiority of the asynchronous condition if the CI lies completely above the noninferiority margin (2%). Weight loss maintenance at 18 and 24 months will be analyzed using the same approach described for aim 1, and we will examine if the CI lies completely above 0 to test if the asynchronous condition has greater weight loss.

For engagement data, we will compare conditions on word count and proportion of the 4 language dimensions (analytical thinking, clout, authenticity, and emotional tone) at 6 months, 1, and 2 years using the same statistical model as described for the primary outcome and test if the resulting CI contains 0. We will graphically explore distributional assumptions and adapt the analyses, if necessary, fitting the most appropriate distribution. We hypothesize that the mean word count per participant will be higher in the asynchronous condition at 1 year and 2 years. Exploratory analyses will compare conditions on the 4 language dimensions at 1 and 2 years as well. Intervention and participant costs per participant will be computed and average costs will be compared across conditions. As Ritzwoller et al [31] recommends, we will perform sensitivity analyses to estimate the range of intervention costs after varying the inputs. We will estimate a range of costs based on varying assumptions. We will compare conditions on total program costs per participant and total program costs per pound lost. Assuming a normal distribution of total costs per participant, we will first compute *t* tests comparing the average cost per participant across treatment conditions. We will test the null hypothesis of no difference between groups using a 2-sided test and α=.05. If the total cost per participant is not normally distributed, a nonparametric approach using the Mann-Whitney test for median comparisons will be used. If participant characteristics are found to differ according to treatment allocation, general or generalized multivariable methods will be used to adjust for the potential confounding effects of these characteristics. Assuming a normal distribution of total costs per participant, multivariable general linear regression models will be used. If not normal, generalized linear models will be used. We hypothesize that the asynchronous condition will cost less per participant (and less per pound lost) than the synchronous condition.

### Ethical Considerations

This trial was approved by the University of Connecticut Human Subjects Institutional Review Board (H23-0383) and is registered on ClinicalTrials.gov (NCT06393725). Possible risks of participating in this trial include exercise-related injury, accidental exposure of personal information, discomfort with study procedures, or an unpleasant interaction with another group member in the group-based intervention. The exercise part of the intervention emphasizes moderate intensity physical activity to reduce the likelihood of discomfort, pain, or injury. The intervention provides guidance on how to do exercises safely to avoid pain or injury. Participants reporting discomfort will be advised to contact their primary care doctor. By excluding people who cannot walk unaided for one-fourth of a mile and those with chronic pain or a medical condition that inhibits their ability to exercise, we reduce the risk of injury. To avoid accidental exposure of personal information, tracking data will be stored electronically in REDCap, a network secure data entry program, and in the UConn-supported R drive which is secure. Only those who have institutional review board approval to work on the study will have access to the R drive and REDCap database for this study. Participants will be informed that they may withdraw from the study at any time if they feel discomfort with any of the study procedures or if they do not want to answer a question on a survey, they can skip it. Participants will be asked to report to their assigned counselor if any comments or conversations in the group make them uncomfortable so that steps can be taken to prevent a recurrence.

## Results

Recruitment will occur from October 2024 to July 2025. Data collection will end on June 2027 and be followed by data analyses. Results will be reported on ClinicalTrials.gov and in a published manuscript.

## Discussion

### Significance of the Study

Obesity affects over 85 million adults in the United States [39]. The workforce needed to make traditional clinic or community-based lifestyle interventions available to all who need them simply does not exist. Intervention approaches that have few barriers to participation and are scalable, sustainable, and low-cost are greatly needed. The rapid shift to telehealth during the COVID-19 pandemic has increased the demand for remote care among the public and health care systems, and necessity has given patients and providers opportunities to learn telehealth skills. The pandemic has also led to a broadening of telehealth reimbursement policies and many of those policies have become permanent [40]. However, reimbursement is limited to synchronous telehealth care (eg, video and calls). Expansion of reimbursement policy to asynchronous care will require evidence for efficacy. Such evidence has been accumulating for behavioral treatments for depression [15,41-43] but is greatly needed for obesity as well. Given how much communication habits have shifted to asynchronous forms (eg, text, email, and social media) in recent years, it is time to examine asynchronous approaches to behavioral interventions. The urgency to produce evidence for innovative telehealth solutions has never been higher and the rapid uptake of telehealth resulting from the pandemic presents opportunities for growth and innovation. Our goal is to produce evidence for an asynchronous lifestyle intervention that is scalable and sustainable.

### Limitations

This study has several limitations. First, lifestyle interventions typically attract predominantly white and female samples [23], which means the literature this study was based on may not be generalizable to more diverse samples. We will use a targeted recruitment approach to attract men and minority populations to prevent this issue. Second, people who are not Facebook users will be excluded, which may also affect the generalizability of the results. Facebook is the most popular social media platform that 68% of US adults report using [44]. The rationale for using Facebook to deliver a lifestyle intervention is to bring content to them on a platform they already use. Studies should explore whether nonusers would be amenable to receiving a lifestyle intervention on Facebook and the barriers that exist for those who prefer not to use Facebook for this purpose. Third, weight data will be collected through digital scales that we ship to participants which prevents us from being physically present when they weigh themselves and ensuring they weigh themselves unclothed and after voiding as instructed. To prevent errant values, we contact participants who lose or gain >5 pounds in a single week or gain ≥5% of their baseline weight at a follow-up visit to verify accuracy and identify the cause if known. By conducting the trial remotely, we can recruit more diverse samples without geographical limitations.

### Conclusions

This work has a high potential for impact in that it could establish evidence that an asynchronous lifestyle intervention that can treat >80 patients at a time is noninferior to a synchronous remote lifestyle intervention in weight loss at both 6 months and 1 year, but superior in sustainability and possibly weight loss maintenance, while also being lower in cost per pound lost. This work will produce a library of 2 years of asynchronous lifestyle intervention content that is suitable for delivery via online platforms and training materials for interventionists and support staff members. This could be used by clinics, large employers, or insurers who could then conduct programs for their target populations. Given the vast ecosystem of patient communities on Facebook, in a future hybrid effectiveness-implementation trial, we plan to partner with leaders of large patient-led Facebook groups to offer this programming to interested members in separate private groups.

## Appendix

Multimedia Appendix 1 Peer-review report by the Lifestyle Change and Behavioral Health Study Section (LCBH) - Risk, Prevention and Health Behavior Integrated Review Group - Center for Scientific Review (National Institutes of Health, USA).

## Abbreviations

DPP: Diabetes Prevention Program
ICC: intraclass correlation coefficient
LIWC-22: Linguistic Inquiry and Word Count
REDCap: Research Electronic Data Capture

## Data Availability

The datasets generated will be available at the NIDDK Central Repository. Data will be available within 1 year after the award ends and be available for 10 years. We will include the DOI in relevant publications.
