# Supplementary material for: Comparing Synchronous and Asynchronous Remotely Delivered Lifestyle Interventions: Protocol for a Randomized Noninferiority Trial
Source: JMIR Res Protoc. 2024 Dec 19;13:e65323. doi: 10.2196/65323 (PMC11695951; doi:10.2196/65323)
Supplement: Multimedia Appendix 1 [file resprot_v13i1e65323_app1.pdf]

SUMMARY STATEMENT
(Privileged Communication)

PROGRAM CONTACT:
Dr. ROBERT KUCZMARSKI
(301) 451-8354
kuczmarskir@niddk.nih.gov

Release Date: 03/02/2023
Revised Date:

Application Number: 1 R01 DK136795-01

Principal Investigator

PAGOTO, SHERRY L.

Applicant Organization: UNIVERSITY OF CONNECTICUT STORRS

Review Group: LCBH
Lifestyle Change and Behavioral Health Study Section

Meeting Date: 02/09/2023
Council: MAY 2023
Requested Start: 07/01/2023
RFA/PA: PA20-183
PCC: NRK OPAT

Project Title: A non-inferiority trial comparing synchronous and asynchronous remotely-delivered lifestyle interventions
SRG Action: Impact Score:32 Percentile:12
Next Steps: Visit https://grants.nih.gov/grants/next_steps.htm
Human Subjects: 30-Human subjects involved - Certified, no SRG concerns
Animal Subjects: 10-No live vertebrate animals involved for competing appl.
Gender: 1U-Both genders, scientifically unacceptable
Minority: 1U-Minorities and non-minorities, scientifically unacceptable
Age: 3U-No children included, scientifically unacceptable

Project Year	
1	[redacted]
2	
3	
4	
5	
TOTAL	

ADMINISTRATIVE BUDGET NOTE: The budget shown is the requested budget and has not been adjusted to reflect any recommendations made by reviewers. If an award is planned, the costs will be calculated by Institute grants management staff based on the recommendations outlined below in the COMMITTEE BUDGET RECOMMENDATIONS section.

1R01DK136795-01 PAGOTO, SHERRY

INCLUSION ACROSS THE LIFESPAN PLAN UNACCEPTABLE
INCLUSION OF MINORITIES PLAN UNACCEPTABLE
INCLUSION OF WOMEN PLAN UNACCEPTABLE

RESUME AND SUMMARY OF DISCUSSION: The overall goal of this application is to determine whether an asynchronous, remote lifestyle intervention is non-inferior to a synchronous, remote lifestyle intervention in weight loss at 6- and 12 months, but more scalable and sustainable, thus producing greater weight loss maintenance at 18- and 24 months among adults with overweight/obesity. The panel agreed on the significance of this project seeking to develop a scalable, asynchronous intervention based on the Diabetes Prevention Program (DPP) that would be more accessible to an underserved population via private Facebook groups and that has the potential to promote weight loss maintenance in the long-term. If successful, the project also has the potential to inform future reimbursement models for behavioral health. The investigative team and research environment are eminently suited for the conduct of this work. The focus on evaluating the asynchronous intervention and on word counts as an engagement metric beyond adherence was highlighted as innovative. Notable strengths in the approach include a well-reasoned RCT study design based on a compelling theoretical model. The panel also acknowledged the preliminary data to support feasibility as a strength. During the discussion, the panel noted weaknesses in the approach. Clinically meaningful weight loss outcomes were not well-supported by previous research demonstrating only 2.6% weight loss. Patient preferences were not sufficiently considered and the lack of attention to the decline in social media usage was not adequately addressed. Although the panel acknowledged that the recruitment plan to include 50% men were laudable, there was a lack of persuasive evidence to support the likelihood this goal would be achieved. Overall, however, the panel concluded that the strengths of this significant application outweigh the weaknesses and the project's potential impact is high.

DESCRIPTION (provided by applicant): Lifestyle interventions have had established efficacy for decades but they are costly and have poor scalability. Remotely delivered lifestyle interventions have increased the potential for scale and systematic reviews have found that they are effective, especially those that include human coaching. Some remote lifestyle interventions are synchronous, such that they are delivered via videoconferencing or phone. Other remote lifestyle interventions are asynchronous, such that they are delivered via online platforms that allow for clinicians and patients to engage via text exchanges or via online groups where patients engage with a clinician and each other by posting messages and contributing to discussion threads. The advantage of asynchronous approaches is 24/7 accessibility which makes them conducive to "just in time" support, allowing patients to engage anytime they want to, as opposed to in scheduled blocks of time each week. Asynchronous, remote interventions may also be more scalable than synchronous remote interventions. We conducted a trial of two asynchronous, remote lifestyle interventions—one with a group of 94 participants and one with a group of 40 participants. Findings revealed similar weight loss and acceptability between conditions. We also found that the larger group was more sustainable, such that participants continued to engage in the group for longer when we turned the groups over to participants to lead themselves for a year after the intervention ended—a period we referred to as the peer-led maintenance phase. The next step in this research is to examine how an asynchronous, remote intervention compares to a synchronous, remote intervention, not only in short term weight loss, but also in sustainability, scalability, and weight loss maintenance. Now that we've established the feasibility of conducing large asynchronous, remote groups, in the proposed trial we will randomize participants to large groups (n=82) in both conditions, which will allow us to compare synchronous to asynchronous remote interventions that are scaled up to a level that we have established is acceptable for asynchronous remote interventions. The purpose of the proposed trial is to determine whether an asynchronous,

remote lifestyle intervention is non-inferior to a synchronous, remote lifestyle intervention in weight loss at 6 and 12 months, but more scalable and sustainable, and thus producing greater weight loss maintenance at 18 and 24 months. Establishing evidence for asynchronous interventions is more important than ever given that telehealth reimbursement for behavioral health has recently expanded but is still limited to synchronous forms of remote care. Asynchronous interventions may be more convenient for some people and possibly more scalable but for this treatment model to reach its potential, evidence for efficacy is needed to inform reimbursement policy. We hypothesize that an asynchronous, remote lifestyle intervention will produce similar initial weight loss as a synchronous, remote version but will be less expensive, more sustainable, and generate greater collective efficacy, which will drive greater weight loss maintenance at 18 and 24 months.

PUBLIC HEALTH RELEVANCE: Traditional lifestyle interventions involve numerous visits for months which is not practical for many people. The proposed randomized trial will compare two telehealth lifestyle interventions, one that is delivered via videoconference meetings and one that is delivered asynchronously in a private Facebook group. Findings will provide evidence for two forms of telehealth-delivered lifestyle interventions.

CRITIQUE 1

Significance: 1
Investigator(s): 2
Innovation: 1
Approach: 3
Environment: 1

Overall Impact: This R01 application seeks to conduct the first non-inferiority RCT (N=328) that will test whether a remotely-delivered lifestyle intervention delivered asynchronously (via private Facebook groups) will result in a mean percent weight loss at 6 and 12 months that is not appreciably worse than one that is delivered synchronously (via teleconferencing). Group size in both conditions will be ~80 participants with implementation taking place in two waves. Measurements will be taken at baseline, 6-, 12-, 18-, and 24-months. Secondary outcomes include retention, and engagement using the novel common metric of word count, cost, and sustainability at 24 months. If successful, the significance of this study is derived in providing the evidence needed to scale asynchronous lifestyle intervention across various platforms in order to reach the millions of Americans who are obese with insufficient scalable cost-effective interventions to reach them. The application is led by a stellar PI with extensive experience and expertise in mHealth and social media-based interventions. The investigation team is quite small but there is a well-resourced and staffed center that is experienced with the trial procedures thus making the timely completion of the study possible. Major strengths of the study include it's novel use of word counts as a common engagement metric between the two study groups, brief training of peer participants to lead the post-active intervention (i.e. maintenance) phase, the cost-effectiveness analyses from the perspectives of program implementation but also the use of a popular and free available social media platform substantially reduces tech development costs. Given the innovation, strength of the approach inclusive of a compelling conceptual model, and strong preliminary data that has significantly informed every aspect of the study resulting in a study that is likely to have a high impact on the field.

1. Significance:

Strengths

- The study will establish the feasibility of conducting a large asynchronous (n=82) weight loss group based on the DPP program to an asynchronous group both hosted remotely thus pushing the field forward as the first study to evaluate the effects of these simultaneous interventions on weight loss
- Asynchronous lifestyle interventions delivered via social media platforms such as Facebook have high potential for scalability given the volume of people who already use these platforms as well as sustainability as long as the groups remain intact which has a greater propensity due to lower costs and time demand than synchronous groups which require scheduling etc.
- If successful, the results of the study may provide sufficient evidence to demonstrate that asynchronous lifestyle interventions can provide comparable results to synchronous interventions delivered at a lower cost which may open the door to reimbursement models for those individuals who are more likely to be responsive to this approach

Weaknesses

- None noted

2. Investigator(s):

Strengths

- The PI (Pagoto) is the Director of the UConn Center for mHealth and Social Media. She is an expert in leveraging technology in behavioral treatments for obesity, specifically using social media platforms to deliver these programs. She also has extensive experience delivering remote and in-person synchronous behavioral weight loss interventions, both clinically and in her research.

Weaknesses

- The only other investigator is the statistician (Huedo). While she has the needed skills and experience in research focusing on obesity prevention, treatment, and weight loss maintenance there does appear to be any previous collaborative studies conducted with the PI or publications

3. Innovation:

Strengths

- Use of a large asynchronous group lends itself to a higher volume of engagement in the maintenance phase
- The use of word count as a common metric of engagement between the two groups is highly novel, and is an extraordinarily meaningful engagement metric because it reflects the time invested by participants and their cognitive engagement in the intervention rather than the common metric of attendance which only reflects showing up which is different from actual engagement
- Comparing Asynchronous vs Synchronous Remote Interventions is Innovative
- This will be the first study to compare asynchronous and synchronous lifestyle interventions in groups where ~80 participants are treated simultaneously.
- The Peer-Led Maintenance Phase is novel and innovative as it will demonstrate whether the program can result in a longstanding online patient community that provides health benefits.

Weaknesses

- None noted

4. Approach:

Strengths

- Conceptual framework is based on Bandura's concept of collective efficacy in which those connected via social media (asynchronous) will establish meaningful relationships among peer groups, and should collaboration occur the members will have the ability to work together to come up with solutions which lends its to the peer maintenance group
- Recruitment for this proposed study will be conducted completely online and through virtual measures such as phone calls, online surveys, and webinar orientations
- After the active activation both groups will enter a peer maintenance phase for another year, thus providing evidence of which intervention arm is most effective for long-term weight maintenance
- To improve sustainability, the investigation team will strengthen the skills of the peer volunteers by providing a brief training on how to lead a group and library of content from the DPP to post in the group as they see fit
- Use of the Facebook platform, which has important usability data among US adults across demographic backgrounds, which is key to generalizability, scalability, and sustainability

Weaknesses

- Like most behavioral interventions studies the investigator's previous studies have been majority female while the categorical age groupings, race and location of users were provided in table 3, this data would be strengthened by also displaying the gender breakdown of Facebook users
- While the PI is aiming for 50% men this has not come to fruition in previous studies even with strategic recruitment strategies (i.e., pay for ads on Reddit) the large synchronous intervention comparison may be even less appealing to men to be in an online group on social media with women.
- The inclusion criteria of being a used who engaged in Facebook at least 5 days per week over the past 2 weeks may exclude participants who could benefit and would engage more for specific content. The justification of 5 vs 4 vs 3 days is not provided. If the intention is for participants to access the content when available to them (ie just in time) a participant can choose to access content on the goal setting days (Monday), weigh in days (Friday) and goal accountability days (Sunday).
- Clarification regarding the exclusion criteria is needed as section 2.2 in the study population characteristics and research design section 3.5.1.6 list different exclusion criteria with the additions of the following three features in the former section "Not interested in losing weight", "Had major surgery in past 6 months" and "Has an implanted cardiac defibrillator or pacemaker." A scientific justification for excluding nicotine users is also warranted, especially in light of the fact that a greater proportion of men tend to be smokers compared to women.

5. Environment:

Strengths

- The UConn Center for mHealth and Social Media, directed by Dr. Sherry Pagoto (PI). The Center leverages mobile technology and social media in the prevention and treatment of disease. The Center has three Cores including: research, technology, and training. Research focuses on the development and evaluation of mobile health apps, social media programs, as well as the use and assessment of biosensing technology on lifestyle behaviors such as weight management, smoking, substance abuse, and physical activity

Weaknesses

- None noted

Study Timeline:

Strengths

- Details the enrollment periods and implementation waves with an adequate study start up period

Weaknesses

- None noted

Protections for Human Subjects:

Acceptable Risks and/or Adequate Protections

Data and Safety Monitoring Plan (Applicable for Clinical Trials Only):

Acceptable

- o The safety officers for the project will include two colleagues independent from the research team with expertise in biostatistics, weight loss interventions, understanding of social media research and of the types and severity of injuries commonly experienced during weight loss trials.

Inclusion Plans:

- Sex/Gender: Distribution not justified scientifically
- Race/Ethnicity: Distribution justified scientifically
- For NIH-Defined Phase III trials, Plans for valid design and analysis: Not applicable
- Inclusion/Exclusion Based on Age: Distribution justified scientifically
- 18-65 years old efficacy literature for study treatments does not include studies of children under 18; 50% men and 30% minorities
- While the PI is aiming for 50% men this has not come to fruition in previous studies even with strategic recruitment strategies (i.e., pay for ads on Reddit) the large synchronous intervention comparison may be even less appealing to men to be in an online group on social media with women.

Vertebrate Animals:

Not Applicable (No Vertebrate Animals)

Biohazards:

Not Applicable (No Biohazards)

Resource Sharing Plans:

Acceptable

Budget and Period of Support:

Recommend as Requested

Recommended budget modifications or possible overlap identified:

- budget outlined in detail.

CRITIQUE 2

Significance: 4
Investigator(s): 1
Innovation: 5
Approach: 4
Environment: 1

Overall Impact: The objectives of this research (R01) are to compare the effectiveness of a synchronously vs asynchronously delivered remote lifestyle intervention promoting weight loss, evaluate program engagement and cost per pound lost, and explore differences in program weight loss maintenance. Facebook will be used for asynchronous intervention delivery and Zoom will be used for synchronous intervention delivery. The trial will consist of a 1-year weight loss phase with content based upon the DPP, and a 1-year peer-led weight loss maintenance phase. A large-group format will be used (80 participants per group, 2 groups per conditions). This is a non-inferiority trial. The investigators are highly-qualified, and the environment provides all of the resources necessary to carry out this research. Additional strengths include many aspects of the approach including the use of DPP content and trained counselors, the RCT design, the large-group format which could increase program access and scalability, and the assessments of participant engagement using several types (likes, text) and aspects (tone) of engagement. Factors that diminish enthusiasm for this research include the limited degree of weight loss in previous asynchronous weight loss interventions (<3%), not considering participant preference for program type, the possible decline in Facebook usage particularly among young adults, the exclusion of those over age 65, and limited degree of innovation. Given these strengths and weaknesses, the potential overall impact on the field is moderate.

1. Significance:

Strengths

- This research will provide information on the effectiveness of asynchronous lifestyle intervention compared to a synchronous approach, which is needed to justify reimbursement for treatment. Asynchronous approaches overcome some participation barriers (e.g., scheduling), and program delivery costs may be lower.
- Most US adults use some form of social media (>70%). This and other types of asynchronous communication (e.g., text messaging, email) are familiar and widely used.

- If successful, the large-group format could increase access to treatment.
- Using social media for intervention delivery could promote continued participation and program maintenance following completion of the intervention.

Weaknesses

- This team previously conducted a trial that included an asynchronous lifestyle intervention arm. Weight loss at 1 year was 2.6% of body weight, which does not quite reach the range considered clinically meaningful (3-5%). Given this degree of weight loss, it is hard to justify a new trial testing this approach.
- Participant preference may impact program effectiveness. In the previous trial, one-third of those interested did not participate due to the time commitment of group visits.
- Facebook usage in the US appears to be declining, particularly among young adults.

2. Investigator(s):

Strengths

- PI Pagoto is Professor in the Department of Allied Health Sciences at the University of Connecticut. She directs the Center for mHealth and social media. Dr. Pagoto is a clinical psychologist whose research is focused on using technology to deliver behavioral weight management programs, including social media-delivered interventions. She has extensive expertise in this area.
- Co-I Huedo-Medina is an Associate Professor at the University of Connecticut, with expertise in biostatistics.
- These investigators are extremely well-qualified to carry out this research.

Weaknesses

- None noted by reviewer

3. Innovation:

Strengths

- Synchronous and asynchronous program delivery will be compared in a large-group format. This has not yet been done. The remote delivery of a program using large group format could increase access to treatment and increase program scalability.
- The 1-year peer-led maintenance phase could improve program sustainability.
- Methods used to assess program engagement were innovative – this includes different types (e.g., likes, text, polls) and aspects (e.g., emotional tone) of engagement.

Weaknesses

- Data presented in table 2 does not support sustained program engagement in a peer-led maintenance program beyond 8 months.

4. Approach:

Strengths

- The RCT design is a strength. Both groups will receive lifestyle counseling – either delivered via private Facebook group or videoconference (Zoom). Intervention content is based upon the DPP.
- The large-group format is a strength. This could minimize program delivery costs, thus potentially increasing access to treatment.
- Participant engagement data will be collected in several ways (text, likes, poll votes; words spoken in the videoconference condition) and assigned a common metric (word counts) using software. Group engagement (collective efficacy, collaboration) will also be evaluated. Higher levels of engagement may support program success.
- Recruitment methods are strong and feasible; this group has reached recruitment goals in previous studies that is comparable to the proposed sample size.
- Counselors will be trained using a CDC-approved DPP Lifestyle Coach Training.
- Body weight data will be collected remotely using cellular scales at months 0, 6, 12, 18, and 24 months.
- Programs costs will be evaluated in order to calculate program costs per pound lost.

Weaknesses

- The upper age limit for participation is age 65. Obesity is common among older individuals. Effective weight management approaches are needed for our aging population. Medical exclusion criteria should be adequate to exclude those who could not safely participate. The CDC's National DPP age eligibility is 18+ years.
- Nicotine users are excluded.

5. Environment:

Strengths

- This research will take place at University of Connecticut's Institute for Collaboration on Health, Intervention, and Policy. This institute includes the Center for mHealth and Social Media, which is directed by the PI. This facility has all of the physical and technological resources needed to carry out the proposed research.

Weaknesses

- None noted by reviewer

Study Timeline:

Strengths

- The timeline is clear and detailed.

Weaknesses

- None noted by reviewer

Protections for Human Subjects:

Acceptable Risks and/or Adequate Protections

Data and Safety Monitoring Plan (Applicable for Clinical Trials Only):

Acceptable

Inclusion Plans:

- Sex/Gender: Distribution justified scientifically
- Race/Ethnicity: Distribution justified scientifically
- For NIH-Defined Phase III trials, Plans for valid design and analysis: Not applicable
- Inclusion/Exclusion Based on Age: Distribution not justified scientifically
- Rationale for excluding those over age 65 is inadequate.

Vertebrate Animals:
Not Applicable (No Vertebrate Animals)

Biohazards:
Not Applicable (No Biohazards)

Resource Sharing Plans:
Acceptable

Budget and Period of Support:
Recommend as Requested

CRITIQUE 3

Significance: 2
Investigator(s): 1
Innovation: 2
Approach: 3
Environment: 1

Overall Impact: Obesity, a significant risk factor for type 2 diabetes, affects 39% of US adults. Lifestyle interventions such as the Diabetes Prevention Program (DPP) have shown strong efficacy for preventing diabetes, but have weak scalability and sustainability. Technology allows us to provide behavioral programs to patients in ways that may be more scalable and sustainable. The effectiveness of synchronous weight loss interventions has been established and thus reimbursable; however asynchronous interventions, which may be more convenient, scalable and sustainable than synchronous interventions, have not been adequately tested. Data regarding asynchronous interventions is needed for future telehealth reimbursement. Drs. Pagoto and Huedo-Medina propose a randomized control trial of 328 participants to test whether a remotely-delivered lifestyle intervention delivered asynchronously via Facebook groups will result in mean percent weight loss at 6 and 12 months that is not appreciably worse than one that is delivered synchronously via teleconferencing. The team has already established the feasibility of their asynchronous Facebook intervention and synchronous teleconferencing intervention. The group size of both conditions will be about 80

participants, which will allow them to compare scaled up versions of these two intervention approaches. In addition to assessment at baseline, 6, and 12 months, 18 and 24 month assessments will measure weight loss maintenance. Weight change, collective efficacy, engagement, sustainability, counselor time, and cost will be assessed. Overall, this is a very strong grant. This proposal is highly significant, with implications for future policy regarding the reimbursement of remotely delivered weight loss interventions. The investigative team is strong, the study is innovative, and the approach is overall strong. The major score-driving weakness is that the team's prior work has only established their ability to recruit predominately female, White samples; despite the recruitment strategies listed, it is doubtful that the team will be able to meet their recruitment benchmark of 50% male and 30% minority, which would limit the study's external validity.

1. Significance:

Strengths

- Asynchronous weight loss interventions may be more convenient, scalable and sustainable than synchronous intervention; however, in contrast to synchronous interventions, there is limited data on the impact of asynchronous interventions on weight loss and maintenance.
- Data on asynchronous weight loss interventions is needed to establish evidence for telehealth reimbursement.
- Testing weight loss strategies that can be scaled up is important for wide-spread dissemination and implementation.

Weaknesses

- None noted by reviewer

2. Investigator(s):

Strengths

- Dr. Pagoto is a premier researcher in leveraging technology in behavioral treatments for obesity
- Dr. Pagoto directs the Center for mHealth and Social Media
- Dr. Huedo-Medina has the necessary expertise in biostatistics

Weaknesses

- Dr. Pagoto and Dr. Huedo-Medina do not have a history of collaboration

3. Innovation:

Strengths

- Examination of a peer-led maintenance phase
- Common engagement of word count

Weaknesses

- None noted by reviewer

4. Approach:

Strengths

- The investigative team have a large body of prior work that clearly justifies the proposed study, with demonstrated feasibility and data regarding their proposed asynchronous weight loss intervention, previous conduct in non-inferiority trials, utilization of Facebook as a platform to deliver lifestyle interventions, lower cost of asynchronous vs. in-person interventions, a larger intervention group size, and a maintenance component.
- Valid and reliable measures
- Strong design, rigorous methods
- Thorough analysis of engagement
- There is an assessment of potential contamination

Weaknesses

- In the team's prior studies, predominately women and Whites were recruited; it is not clear that they will be able to recruit 50% men and at least 30% minorities.
- It is not clear why a cut-off of BMI 27 was used instead of 25
- Is the Counselor the Interventionist? This is not clear.

5. Environment:

Strengths

- The Center for mHealth and Social Media is a critically important and valuable resource
- InCHIP provides all of the facilities needed to execute the proposed project

Weaknesses

- None noted by reviewer

Study Timeline:

Strengths

- Comprehensive and realistic

Weaknesses

- None noted by reviewer

Protections for Human Subjects:

Acceptable Risks and/or Adequate Protections

Data and Safety Monitoring Plan (Applicable for Clinical Trials Only):

Acceptable

Inclusion Plans:

- Sex/Gender: Distribution not justified scientifically
- Race/Ethnicity: Distribution not justified scientifically
- For NIH-Defined Phase III trials, Plans for valid design and analysis: Not applicable
- Inclusion/Exclusion Based on Age: Distribution justified scientifically

- Despite the recruitment strategies listed, it is doubtful that the team will be able to meet their recruitment benchmark of 50% male and 30% minority

Vertebrate Animals:

Not Applicable (No Vertebrate Animals)

Biohazards:

Not Applicable (No Biohazards)

Resource Sharing Plans:

Acceptable

Budget and Period of Support:

Recommend as Requested

THE FOLLOWING SECTIONS WERE PREPARED BY THE SCIENTIFIC REVIEW OFFICER TO SUMMARIZE THE OUTCOME OF DISCUSSIONS OF THE REVIEW COMMITTEE, OR REVIEWERS' WRITTEN CRITIQUES, ON THE FOLLOWING ISSUES:

PROTECTION OF HUMAN SUBJECTS: ACCEPTABLE

INCLUSION OF WOMEN PLAN: UNACCEPTABLE
Although the panel acknowledged that the recruitment plan to include 50% men was laudable, there was a lack of compelling evidence to support the likelihood this goal would be achieved.

INCLUSION OF MINORITIES PLAN: UNACCEPTABLE
Additional consideration and justification could be given for the recruitment of minorities.

INCLUSION ACROSS THE LIFESPAN PLAN: UNACCEPTABLE
The scientific rationale for excluding participants over age 65 was considered inadequate.

COMMITTEE BUDGET RECOMMENDATIONS: The budget was recommended as requested.

Footnotes for 1 R01 DK136795-01; PI Name: PAGOTO, SHERRY L.

NIH has modified its policy regarding the receipt of resubmissions (amended applications).See Guide Notice NOT-OD-18-197 at https://grants.nih.gov/grants/guide/notice-files/NOT-OD-18-197.html. The impact/priority score is calculated after discussion of an application by averaging the overall scores (1-9) given by all voting reviewers on the committee and multiplying by 10. The criterion scores are submitted prior to the meeting by the individual reviewers assigned to an application, and are not discussed specifically at the review meeting or calculated into the overall impact score. Some applications also receive a percentile

ranking. For details on the review process, see http://grants.nih.gov/grants/peer_review_process.htm#scoring.
